# Association Between Vitamin D and Diabetic Kidney Disease

**DOI:** 10.3390/jcm15010153

**Published:** 2025-12-25

**Authors:** Feride Pınar Altay, Özlem Turhan İyidir, Sevim Güllü

**Affiliations:** 1 Endocrinology and Metabolic Diseases, Ankara Bilkent City Hospital, Ankara 06800, Turkey; 2 Endocrinology and Metabolic Diseases, Faculty of Medicine, Başkent University, Ankara 06490, Turkey; oturhaniyidir@yahoo.com; 3 Endocrinology and Metabolic Diseases, Faculty of Medicine, Ankara University, Ankara 06230, Turkey; sevim.gullu@temd.org.tr

**Keywords:** diabetic kidney disease, vitamin D, microalbuminuria

## Abstract

**Background:** Diabetic kidney disease (DKD) is the most important cause of end-stage renal failure. The aim of this study is to investigate whether there is an association between supplementation of vitamin D and DKD or not. **Methods**: The study was designed prospectively and initiated with a total of 81 patients with a history of type 2 diabetes mellitus (DM) and diagnosed with stage 3 or 4 diabetic nephropathy (DN), who applied to Ankara University Faculty of Medicine between July 2011 and February 2013. It was completed with a total of 63 patients, 38 female (60.3%) and 25 male (39.7%), during the six-month follow-up period. The inclusion criteria were as follows: microalbumin ≥ 30 mg/day in 24 h urine, for which at least two measurements were obtained; age ≥ 18; HbA1c ≤ 8%; eGFR (estimated glomerular filtration rate) ≥ 30 mL/min; and, in addition, type 2 DM diagnosis. Patients with microalbumin levels of 30–299 mg/24 h were included in the microalbuminuria group, whereas patients with ≥300 mg were included in the macroalbuminuria group. An oral dose of 300,000 IU vitamin D3 replacement was given to patients with vitamin D deficiency and insufficiency. **Results**: In both groups, a significant increase in vitamin D levels at six months compared to baseline was observed, while a significant decrease in 24 h urine microalbumin and protein levels was observed at six months. Considering these results, vitamin D was considered to have a positive effect on 24 h urine microalbumin and protein levels. **Conclusions**: In both groups, a significant increase in vitamin D levels and a significant decrease in microalbumin and protein levels were detected at the sixth month via 24 h urine tests. Therefore, vitamin D replacement is thought to be beneficial for DKD treatment because of its antiproteinuric effect.

## 1. Introduction

Diabetes is one of the most significant health problems faced worldwide, and its prevalence has increased from an estimated 30 million in 1985 to 285 million in 2010 [1]. Based on current trends, the International Diabetes Federation (IDF) projects that there will be 438 million people with diabetes by 2030 [2]. The Centers for Disease Control and Prevention (CDC) estimates that 25.8 million people, or 8.3% of the United States population, had diabetes in 2010 [3].

Similarly, Turkish Diabetes Epidemiology Study II (TURDEP II) showed that diabetes prevalence in Turkey was13.7% of the population in 2010 and has increased by 90% in the last 12 years [4].

DKD occurs in 20–40% of patients with diabetes and it is the most important cause of end-stage renal failure [5]. According to 2009 data from the Turkish Society of Nephrology, 35% of patients with end-stage renal failure in Turkey have diabetes mellitus [6].

Although not fully defined, genetic, hemodynamic, and metabolic factors, and various intracellular signaling molecules, growth factors, and cytokines initiated by hyperglycemia are classified as responsible for the pathogenesis of diabetic complications.

The renin–angiotensin system (RAS) plays a key role in the pathogenesis of DN. Over-activation of the RAS leads to renal and cardiovascular disorders. Epidemiological and clinical studies have shown that vitamin D deficiency or insufficiency is associated with an increased risk of renal and cardiovascular diseases. Vitamin D plays a role in the negative endocrine control of the RAS. 1.25-Dihydroxyvitamin D3 (1.25(OH)2D3) reduces and regulates renin expression. Vitamin D deficiency leads to the over-expression of renin, which prompts activation of the RAS, causing renal and cardiovascular injuries. The suppression of the RAS by vitamin D is thought to protect renal and cardiovascular homeostasis. Vitamin D analogs can be used in the treatment of renal and cardiovascular diseases [7].

Increased intra-renal renin and angiotensinogen levels have been determined in diabetic mice [8,9]. Furthermore, intra-renal RAS is known to play a role in renal injury.

Renin expression and plasma angiotensin II production were found to be increased in vitamin D receptor-null (VDR-null) mice, leading to hypertension, cardiac hypertrophy and increased water intake [10]. Active vitamin D metabolites bind to the vitamin D receptor (VDR) and inhibit the stimulation of the RAS as well as podocyte and mesangial cell proliferation. In addition, active vitamin D prevents fibrogenesis by inhibiting TGF-β indirectly. As a result, active vitamin D metabolites reduce glomerulosclerosis and tubulointerstitial fibrosis [11]. ACEI, ARB and renin inhibitors, which are used for treatment of DN, cause RAS inhibition. By using these therapies, the progression of glomerulosclerosis, and tubulointerstitial fibrosis and proteinuria, is reduced. However, the main problem is that these medications cause compensatory renin increases. An important application of vitamin D analogs is that they block compensatory renin increases at the transcriptional level when used in conjunction with classical RAS inhibitors [7].

In this study, it is thought that vitamin D may be useful in the treatment of DKD in the light of the studies mentioned above.

## 2. Materials and Methods

The prospective study was initiated with total of 81 patients with history of both type 2 DM and stage 3 or 4 DN, who applied to Ankara University School of Medicine between July 2011 and February 2013, but was completed with 38 female (60.3%), 25 male (39.7%), and a total of 63 patients. That is, 18 patients (22%) had to be excluded from the follow-up because they did not come for the 6-month follow-up during the study.

The inclusion criteria include the following: microalbumin ≥ 30 mg/day in 24 h urine, which is obtained at least at two measurements; age ≥ 18; HbA1c ≤ 8%; eGFR (estimated glomerular filtration rate) ≥ 30 mL/min; and, in addition, type 2 DM diagnosis. Patients with microalbumin of 30–299 mg/24 h were included in microalbuminuria group, whereas those with ≥300 mg included in macroalbuminuria group.

The American Diabetes Association (ADA)’s criteria have been taken into account for diagnostic of type 2 diabetes. As a first step, the background information of all patients has been collected along with their medical history. The physical diagnostics of patients were recorded during the six-month screening period. Blood pressures were measured and recorded with the same sphygmomanometer after a 15 min rest and when the patients were in a sitting position. The body mass index (BMI) values of all patients were calculated when first introduced to the study and then six months later. The patients’ FPG, 2h-PG, HbA1c, fasting insulin, creatinine, ALT, Ca, P, albumin, PTH, vitamin D, total cholesterol, LDL cholesterol, HDL cholesterol, triglyceride, and microalbumin, protein, and creatinine in 24 h urine were studied.

Patients with microalbumin of 30–299 mg/24 h were included in microalbuminuria group, whereas those with ≥300 mg included in macroalbuminuria group. An oral dose of 300,000 IU vitamin D3 replacement was given to patients with vitamin D deficiency and insufficiency. In both groups, the association between the baseline and sixth-month vitamin D value and microalbumin, and protein in 24 h urine has been studied. Clinical and biochemical parameters that can be related to type 2 DM and DN were evaluated, too.

In order to evaluate renal functions, the below given expressions (1)–(3) have been used.

(1) $$CC\left(mL/min\right):\frac{urine\;creatinine\left(\frac{mg}{dL}\right)\times urine\;volume\;(mL)}{Blood\;creatinine\;\left(\frac{mg}{dL}\right)\times 1440}$$

(2) $${eGFR}_{MDRD}\left(\frac{mL/min}{1.73m^{2}}\right):186\times {Serum\;creatinine}^{-1.154}\times {age}^{-0.203}\times \left(0.742\;if\;female\right)$$

(3) $${eGFR}_{Cockcroft}\left(mL/min\right):\frac{\left(140-age\right)\times weight\left(kg\right)\times (0.85\;if\;female)}{72\times serum\;creatinine\;(mg/dL)}$$

On the other hand, for evaluating the insulin resistance, expression (4) has been used.

(4) $$HOMA\;IR:\frac{APG\;(mg/dL)\times insulin\;(\mu U/mL)}{405}$$

## 3. Statistical Evaluation

SPSS 15.0 was used for statistical analysis in the evaluation of the obtained data. Variables with a normal statistical distribution were presented as mean ± standard deviation, and variables without a normal distribution were presented as median (min–max). The number of cases and percentages were used for nominal parameters. If the changes in the variables over time were normally distributed, the paired *t*-test was used; otherwise, the Wilcoxon test was used.

The significance of differences between groups for mean values was assessed using Student’s *t*-test, and for median values, the Mann–Whitney U test was used. Nominal variables were evaluated using Pearson’s chi-square or Fisher’s exact test. The Spearman correlation test was used to examine the relationship between continuous variables when the distribution was not normal, and Pearson’s correlation test was used when the distribution was normal.

Multiple linear regression analysis was performed to determine the independent factors affecting microalbuminuria variation. The results were evaluated, and values with a probability of *p* < 0.05 were considered statistically significant. Bonferroni correction was applied when necessary.

## 4. Results

A total of 63 patients were included in the study: 38 females and 25 males. The mean age of the patients was 57.92 ± 9.8 years. In total, 54 patients were in the microalbuminuria group and 9 were in the macroalbuminuria group. Fifteen patients with microalbuminuria and three with macroalbuminuria were excluded from the study due to the absence of follow-up. Demographic characteristics, blood pressure, and the treatment of the patients in both groups are presented in Table 1.

As seen in Table 1, there is no significant difference between the demographic characteristics, blood pressure, and treatment of the patients, other than the insulin therapy received by the patients in both groups. The insulin usage in the macroalbuminuria group is higher than that of the microalbuminuria group (*p* = 0.009). Table 2 indicates baseline biochemical values of patients.

When the baseline biochemical values of patients were evaluated, the vitamin D values in macroalbuminuria group were found to be lower than that of the microalbuminuria group (*p* = 0.022). On the other hand, the 2h-PG (*p* = 0.01), cystatin C (*p* = 0.017), Microalbuminuria/24 h (*p* = 0.000), and proteinuria/24 h (*p* = 0.014) values were significantly higher in macroalbuminuria group compared with microalbuminuria group.

Table 3 indicates the baseline and sixth-month clinical parameters of the microalbuminuria group.

When the baseline and sixth-month clinical parameters of the microalbuminuria group given in Table 3 were considered, meaningful significant differences in the vitamin D (*p* = 0.006), microalbuminuria/24 h (*p* = 0.001), proteinuria/24 h (*p* = 0.013), HDL (*p* = 0.036), PTH (*p* = 0.001), and P (*p* = 0.019) values have been found.

**Figure 1:**
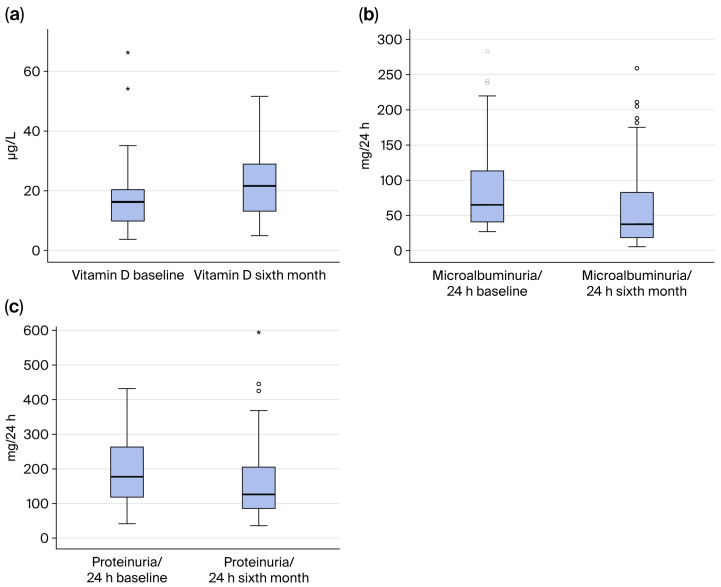
(**a**) Vitamin D variation in the microalbuminuria group at baseline and at the sixth month. (**b**) Microalbuminuria/24 h variation in microalbuminuria group at baseline and at the sixth month. (**c**) Proteinuria/24 h variation in microalbuminuria group at baseline at the sixth month.

Figure 1 indicates vitamin D, microalbuminuria/24 h, and proteinuria/24 h variation in the microalbuminuria group at baseline and after six months.

Figure 1a, shown above, indicates a significant increase in vitamin D values at the sixth month compared with baseline values (*p* = 0.006). Figure 1b indicates microalbuminuria/24 h variation in the microalbuminuria group at baseline and at the sixth month. As seen from this figure, there is a significant decrease in microalbuminuria/24 h at the sixth month compared with the baseline (*p* = 0.001). Figure 1c indicates proteinuria/24 h variation in the microalbuminuria group at baseline and at the sixth month. As seen from this figure, there is a significant decrease in proteinuria/24 h at the sixth month compared with the baseline value (*p* = 0.013).

In this group, a meaningful increase has been defined at the sixth-month HDL levels when compared with the baseline values (*p* = 0.036). In addition, a decrease in the sixth-month PTH and P values has been identified (*p* = 0.001, *p* = 0.019, respectively).

Table 4 indicates baseline and sixth-month clinical parameters of macroalbuminuria group.

**Figure 2:**
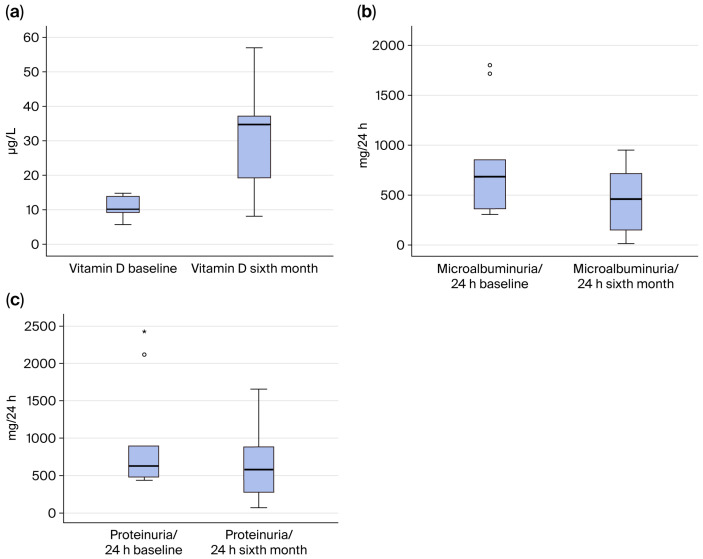
(**a**) Vitamin D variation in the macroalbuminuria group at baseline and at the sixth month. (**b**) Microalbuminuria/24 h variation in macroalbuminuria group at baseline and at the sixth month. (**c**) Proteinuria/24 h variation in macroalbuminuria group at baseline and at the sixth month.

Figure 2 shows changes in vitamin D, microalbuminuria/24 h, and proteinuria/24 h at baseline and after six months in the macroalbuminuria group.

Figure 2a, shown above, indicates a significant increase in vitamin D values in the sixth month compared with the baseline values (*p* = 0.004). Figure 2b indicates microalbuminuria/24 h variation in the macroalbuminuria group at baseline and at the sixth month. As seen from this figure, there is a significant decrease in microalbuminuria/24 h inthe sixth month compared with the baseline value (*p* = 0.021). Figure 2c indicates proteinuria/24 h variation in macroalbuminuria group at the baseline and in the sixth month. As seen from this figure, there is a significant decrease in proteinuria/24 h in thesixth month compared with baseline value (*p* = 0.028).

A meaningful decrease has been seen in the sixth-month eGFR_MDRD_ and eGFR_CockCroft_ values compared with baseline in the macroalbuminuria group (*p* = 0.028, *p* = 0.046, respectively). It was defined that although the vitamin D increase was more obvious, the microalbuminuria/24 h change was less in the macroalbuminuria group.

No significant correlation was found between percent change in vitamin D and percent change in either the microalbuminuria/24 h or proteinuria/24 h when the baseline and sixth-month values were used in both groups. In the microalbuminuria group, for the microalbuminuria/24 h, *r* = 0.175 and *p* = 0.207, and for the proteinuria/24 h, *r* = 0.076 and *p* = 0.594 values were found. On the other hand, in the macroalbuminuria group, for the microalbuminuria/24 h, *r* = −0.200 and *p* = 0.704, and for the proteinuria/24 h, *r* = −0.179 and *p* = 0.702 values were found.

The regression analysis performed on 63 patients revealed that having a smoking habit increased microalbumin values in 24 h urine by 123.68 times (beta coefficient: 0.269). On the other hand, a one-unit increase in HbA1c increases microalbumin values in 24 h urine by 48.9 times (beta coefficient: 0.246). There was no correlation among percentage changes in LDL, HOMA-IR, and BMI.

When the patients were divided into two groups, as those with vitamin D values below 20 and with those with values above 20, no significant difference was found in 24 h urine microalbumin and protein percentage changes.

In the microalbuminuria group, the percentage change in the microalbumin values in 24 h urine was *p* = 0.224, and the percentage change in the protein values in 24 h urine was *p* = 0.804. In the macroalbuminuria group, the percentage change in microalbumin values in 24 h urine was *p* = 0.857, and the percentage change in the protein values in 24 h urine was *p* = 0.548.

## 5. Discussion

DKD is the most important cause of end-stage renal failure. Both microalbumin and macroalbumin in diabetic patients are associated with increased risk of cardiovascular diseases [1].

Vitamin D takes a role in negative endocrine control in the renin angiotensin system (RAS). RAS has an important role in the pathogenesis of DKD. The over-activation of RAS causes renal and cardiovascular disorders [7].

Increased levels of renin and angiotensinogen have been shown in diabetic rats [8,9]. It is also known that intra-renal RAS plays an important role in renal injury induced by hyperglycemia in diabetes mellitus [12]. Invitro studies indicate that hyperglycemia increases the renin angiotensin production in mezengial cells and podosits [13,14,15]. Angiotensin II in the kidneys provides progression of renal failure [16,17].

Active vitamin D binds to the vitamin D receptor and inhibits mezengial cell proliferation by RAS activation. It also prevents fibrosis by indirectly inhibiting TGF β1 and decreases glomerulosclerosis and tubulointerstitial fibrosis [18].

When taking into account the positive effects of vitamin D on RAS shown in earlier studies, it is thought that vitamin D might be helpful for the treatment of DKD. In line with this consideration, the effects of vitamin D on diabetic nephropathy patients have been investigated in our study.

In the study, 63 of 81 patients, who completed a six-month follow-up period at the Ankara University School of Medicine between July 2011 and February 2013, have been included in the microalbuminuria and macroalbuminuria groups. The changes in the baseline and six-month clinical parameters that were given in Section 4 have been evaluated in both groups.

The major finding of our study is that vitamin D supplementation was associated with significant improvements in several clinical parameters among patients with microalbuminuria. When the baseline and six-month clinical parameters of microalbuminuria group given in Table 3 were considered, meaningful significant differences in vitamin D (*p* = 0.006), microalbuminuria/24 h (*p* = 0.001), proteinuria/24 h (*p* = 0.013), HDL (*p* = 0.036), PTH (*p* = 0.001), and P (*p* = 0.019) values have been found.

However, no significant correlation was identified between the percentage change in vitamin D levels and the percentage change in either 24 h microalbuminuria or proteinuria in both groups when the baseline and six-month values were analyzed.

These findings are in line with previous studies that reported the beneficial renal effects of vitamin D in diabetic patients. Huang et al. demonstrated that daily supplementation with 800 IU cholecalciferol led to a reduction in microalbuminuria in the early stages of treatment compared with the control group [19]. In that study, the baseline vitamin D values in the macroalbuminuria group were lower when compared with those of the microalbuminuria group. This result is consistent with our finding that the baseline vitamin D levels were lower in patients with macroalbuminuria.

Similarly, Kim et al. reported that cholecalciferol at 40,000 units weekly can reduce proteinuria in patients with diabetes [20]. Zhang et al. showed that combination therapy with an AT1 blocker and vitamin D analogs better reduced albuminuria when compared with only AT1 blocker treatment in experimental models [21]. The VITAL study indicated similar results by using combined therapy with paricalcitol and RAS inhibition in diabetic nephropathy patients [22].

Although a significant correlation between the degree of vitamin D increase and proteinuria reduction was not observed, the observed within-group improvements suggest a possible beneficial association rather than a direct causal relationship. Further studies with controlled designs are needed to verify this relationship.

In our study, a meaningful increase in HDL values in the sixth month in the microalbuminuria group was noted (*p* = 0.036). Many studies indicated a positive relationship between vitamin D and HDL cholesterol. Vitamin D is the basic element necessary for the production of apoliprotein A1, which is the main component of HDL cholesterol [23]. Vitamin D can indirectly affect lipid metabolism by increasing insulin sensitivity [24].

In addition, a decrease in sixth-month PTH and *p*-values has been identified (*p* = 0.001, *p* = 0.019, respectively). The increase in vitamin D resulted in a decrease in PTH.

Similar results have been obtained from the macroalbuminuria group with alesser number of patients (n = 9). The increase in sixth-month vitamin D values compared with the baseline has been identified (*p* = 0.004). A meaningful decrease in microalbuminuria and protein in 24 h urine in the sixth month has been seen (respectively, *p* = 0.021; *p* = 0.028).

A meaningful decrease has been seen in sixth-month eGFR_MDRD_ and eGFR_CockCroft_ values compared with baseline in the macroalbuminuria group (*p* = 0.028, *p* = 0.046, respectively). There was decrease in creatinine clearance, but this was not meaningful. The reason behind the lesser decrease in 24 h urine microalbuminuria release in the macroalbuminuria group was thought to be because of the decrease in eGFR.

A significant difference was observed between the baseline cystatin C values of the macroalbuminuria and microalbuminuria groups (*p* = 0.017). Cystatin C levels were considerably higher in the macroalbuminuria group, indicating more advanced renal impairment, while cystatin C concentrations in the microalbuminuria group were also above the normal range. In contrast, serum creatinine levels did not differ significantly between the two groups. These findings suggest that cystatin C may serve as a more sensitive biomarker for early renal dysfunction than serum creatinine.

Previous studies have supported the diagnostic value of cystatin C in diabetic nephropathy. Willems et al. indicated that cystatin C was a more sensitive parameter in the diagnosis of early-stage diabetic nephropathy when compared with creatinine [25]. Similarly, Jean et al. revealed that cystatin C was a useful indication of early renal injury in normoalbuminuric patients having type 2 diabetes [26]. It was shown by Lopez et al. that cystatin C levels were able to be used as early diagnosis of vascular and renal injury [27]. However, another study indicated that cystatin C was not superior to creatinine [28].

In addition, Suzuki et al. found that cystatin C levels were increased in patients with microalbuminuria, whereas serum creatinine and β2-microglobulin levels showed no remarkable elevation. They concluded that cystatin C offers greater sensitivity and specificity when detecting early diabetic nephropathy compared with these traditional markers [29]. Moreover, both albuminuria and reduced GFR are known risk factors for mortality in diabetic patients. A previous study [30] indicated that GFR estimated using cystatin C provides a more accurate prediction of mortality risk than GFR calculated based on serum creatinine.

Our findings in the macroalbuminuria group were directionally similar but more limited, possibly due to the small number of participants (*n* = 9) and more advanced renal impairment, as reflected by significantly elevated cystatin C levels. Cystatin C has been reported as a more sensitive marker of early renal dysfunction than creatinine [25,26,27,28,29]. The current results support this, as higher cystatin C values in the macroalbuminuria group indicate greater renal injury, which could also attenuate the response to vitamin D supplementation.

In our study, NT-proBNP levels did not differ significantly between the microalbuminuria and macroalbuminuria groups (*p* = 0.953). Although the NT-proBNP values tended to be higher in patients with macroalbuminuria and in those with a history of coronary artery disease, these differences did not reach statistical significance. This lack of difference may be partly attributed to the small number of patients in the macroalbuminuria subgroup, which limits its statistical power.

Previous studies have shown that NT-proBNP is an important biomarker reflecting both cardiac and renal dysfunction in diabetes. Beer et al. reported that NT-proBNP levels were markedly elevated in patients with micro- or macrovascular complications compared with those without such complications [31]. Similarly, Danış et al. found that NT-proBNP levels were higher in diabetic patients with macroalbuminuria than in those with microalbuminuria or normoalbuminuria, suggesting its potential role as an early marker of diabetic nephropathy [32]. Furthermore, NT-proBNP concentrations have been shown to correlate with increased cardiovascular risk and mortality in diabetic patients [33,34].

In the context of our findings, the absence of a significant difference in NT-proBNP between the albuminuria groups should therefore be interpreted with caution. It may reflect the limited sample size, relatively short follow-up, and possible overlap of renal and cardiac dysfunction among participants rather than the absence of a biological relationship.

Chronic hyperglycemia is the main cause for pathogenesis of diabetic nephropathy. The effects of vitamin D on insulin secretion and insulin sensitivity are well known. Vitamin D affects beta cells more than alfa cells and increases insulin secretion as a response to glucose stimulation [18,35].

It was shown that insulin secretion was reduced in rats with vitamin D deficiency [36], and impaired glucose tolerance and insulin secretion were better after single-dose vitamin D subcutaneous injection [37].

Vitamin D replacement in IGT and DM patients with vitamin deficiencies leads to better insulin secretion, glucose tolerance, and HbA1c [38,39]. Studies show that a decrease in HbA1c reduces microalbuminuria development [40,41].

The positive effects of vitamin D on glycemic control and insulin resistance that are shown in studies have given rise to the ideas regarding the encouraging effects of vitamin D on diabetic nephropathy patients. There was no significant change in FPG, 2h-PG, HbA1c, and fasting insulin values.

A meaningful correlation has not been identified between vitamin D percentage change and both microalbuminuria/24 h and proteinuria/24 h variation in percentage in both groups when baseline and sixth-month values were utilized. It was thought that an increased number of patients may provide more reasonable results.

The regression analysis performed on the 63 patients revealed that having a smoking habit increases microalbumin values in 24 h urine by 123.68 times (beta coefficient: 0.269). Similarly, Gambaro et al. found cigarette smoking as a risk factor for diabetic nephropathy [42]. Kathryn et al. showed that smoking cigarettes until diabetes diagnosis (packs/year) was independently related to microalbuminuria prevalence when HbA1c, age, blood pressure, and the duration of diabetes were under control [43].

The study performed by Chakkarwar et al. indicated that smoking could increase oxidative stress, TGFβ upregulation, and the end products of glycosylation. Cigarette smoking in diabetic patients with vascular complications causes the thickening of the glomerular basement membrane and mesangial expansion with progression in glomerulosclerosis and interstitial fibrosis, which results in end-stage renal failure [44].

The regression analysis that has been performed on our study data reveals that one-unit increase in HbA1c increases microalbumin values in 24 h urine by 48.9 times (beta coefficient: 0.246).

In our study, the effect of vitamin D was evaluated by keeping HbA1c levels under control, since hyperglycemia plays an important role in pathogenesis.

In this study, examining the effects of conventional and intensive insulin treatment on the development risk of chronical complications of diabetes by studying 1441 type 1 diabetic patients in a DCCT study, it is shown that continuously becoming better on HbA1c decreases the risk of development of diabetic neuropathy [40]. Similarly, it is seen that glycemic control is effective for preventing microalbuminuria in patients diagnosed with new type 2 diabetes in a UKPDS study [41].

HbA1c level is higher in patients with microalbuminuria and macroalbuminuria when compared with patients with normoalbuminuria [45]. The regression analysis revealed that there was no meaningful relation with LDL, HOMA-IR, and BMI, which are the other parameters that affect microalbuminuria excretion in 24 h urine.

Study [46] showed that VDD (vitamin D deficiency) or vitamin D metabolism disorder is positively associated with the severity of renal injury. The mechanisms may include vitamin D metabolism disorder, metabolic suppression, insulin resistance, and the abnormal regulation of the immune system through upregulation of inflammatory signaling pathways.

Wang et al. demonstrated the significant role of serum vitamin D levels in influencing the risk of diabetic kidney disease and overall survival in type 2 DM patients [47].

Based on enrolled 328 patients with confirmed diabetes, low vitamin D levels were associated with both type 2 DM and diabetic nephropathy and those patients with type 2 DM were more likely to develop kidney disease [48].

Reviewed studies [49] emphasize the possible roles of vitamin D beyond calcium–phosphate homeostasis modulation in diabetic kidney disease. Experimental studies, observational studies, and clinical trials have indicated the possible effects of vitamin D in protecting against the progression of diabetic kidney disease and preserving the integrity of the glomerular filtration barrier.

Study [50] included 7722 patients and revealed that vitamin D was significantly lower in DN patients compared to diabetic patients without nephropathy. When diabetic patients with normoalbuminuria, microalbuminuria, and macroalbuminuria were compared, vitamin D levels were found to decrease as the disease progressed.

A prospective study included 14,709 participants with type 2 diabetes who were free of microvascular complications, and found that higher serum 25(OH)D concentrations were significantly associated with lower risk of diabetic microvascular complications, including diabetic retinopathy, diabetic nephropathy, and diabetic neuropathy [51].

In a Korean study [52] with 1392 patients with type 2 DM, vitamin D deficiency was common among Korean type 2 DM patients; it was independently associated with microalbuminuria and the HDL level, and positively related to diabetic nephropathy.

He at al. used two-sample Mendelian randomization to examine the causal influence of vitamin D on diabetic nephropathy in 7751 individuals with type I diabetes-related nephropathy and 9933 individuals with type II diabetes-related nephropathy. They indicated that 25(OH)D might reduce the risk of DN, either for type 1 DN or for type 2 DN in the early stage [53].

A total of 4284 Chinese patients with type 2 diabetic mellitus were enrolled into a cross-sectional study [54]. Vitamin D deficiency was significantly associated with a higher prevalence of diabetic foot ulcers among Chinese patients. The association between vitamin D deficiency status and diabetic retinopathy or diabetic kidney disease was not significant when adjusting for all potential covariates.

Another cross-sectional study on 1576 individuals with DM revealed an association between vitamin D levels, HbA1c, and DKD. Additionally, the data suggest that the association between urinary albumin excretion and vitamin D levels is independent of glycemic control in patients with diabetes [55].

Our study contributes to the literature by being prospectively designed, including patients with HbA1c ≤ 8, and by prospectively evaluating numerous clinical and laboratory data.

Because studies on this topic are largely experimental, evaluating the relationship between vitamin D and diabetic kidney disease in patients is valuable in the literature. The most important finding of our study is that vitamin D supplementation is associated with significant improvements in various clinical parameters in patients with microalbuminuria. In light of these findings, vitamin D replacement may be beneficial in diabetic kidney disease.

Our study has some limitations that should be acknowledged. The observational design without randomization or a placebo control group prevents its ability to establish a causal relationship between vitamin D supplementation and changes in proteinuria. The concomitant use of RAS inhibitors and other antihypertensive agents may also have influenced the results. In addition, the small number of patients in the macroalbuminuria group reduces the statistical power and limits the generalizability of the findings.

## 6. Conclusions

A significant increase in vitamin D levels was found compared to baseline values at month six in both the microalbuminuria and macroalbuminuria groups (*p* = 0.006, *p* = 0.004, respectively). A significant decrease in microalbumin and 24 h urine protein levels was found compared to the baseline values at month six in both the microalbuminuria and macroalbuminuria groups (*p* = 0.001, *p* = 0.013, microalbuminuria group; *p* = 0.021, *p* = 0.028, respectively). Considering these results, it is thought that vitamin D may have a positive effect on 24 h urine microalbumin and protein levels.

In conclusion, the significant increase in vitamin D levels observed in both the microalbuminuria and macroalbuminuria groups at the sixth month, and the significant decreases in 24 h urine microalbumin and protein levels in both groups in the sixth month, suggest that vitamin D replacement is beneficial in the treatment of diabetic kidney disease, with an antiproteinuric effect.

## Figures and Tables

**Table 1:** Demographic properties, blood pressure, and treatment of patients.

Variables	Microalbuminuria	Macroalbuminuria	*p*-Value
Gender (F/M)	54 (31/23)	9 (8/1)	0.135
Age	57.30 ± 9.97	61.67 ± 8.74	0.221
SBP (mmHg)	130 (109–170)	140 (125–160)	0.074
DBP (mmHg)	80 (60–100)	80 (70–100)	0.76
BMI (kg/m^2^)	31.95 (22.60–49.90)	36.30 (25.40–44.30)	0.080
OAD	51	7	0.201
Insulin	11	6	0.009
ACEI	17	4	0.466
ARB	28	5	1.00
CaBlc	15	2	1.00
ASA	16	5	0.146
Statin	22	7	0.068
Fenofibrate	1	2	0.051
Smoking	12	1	0.671

Mean ± standard deviation; median (min–max).

**Table 2:** Baseline biochemical values of patients.

Variables	Microalbuminuria	Macroalbuminuria	*p*-Value
HbA1c (%)	6.7 (5.3–8)	7.6 (6–8)	0.157
FPG (mg/dL)	117 (72–227)	136 (93–210)	0.341
2h-PG (mg/dL)	173.5 (83–363)	217 (150–323)	0.01
Fasting insulin (µIU/mL)	13 (2.2–61.8)	11.1 (5.8–28.8)	0.761
LDL (mg/dL)	112.5 (53–238)	99 (75–187)	0.562
HDL (mg/dL)	42 (26–68)	42 (33–64)	0.536
TG (mg/dL)	128.5 (35–489)	146 (91–331)	0.235
Cr (mg/dL)	0.85 (0.45–1.87)	0.89 (0.62–1.39)	0.331
ALT (U/L)	19 (8–70)	21 (17–38)	0.270
Vitamin D (µg/L)	16.25 (3.7–66.4)	10.2 (5.8–14.8)	0.022
Ca (mg/dL)	9.4 (8.5–10.2)	9.6 (9.3–10.4)	0.091
P (mg/dL)	3.71 ± 0.56	3.64 ± 0.35	0.094
Alb (g/dL)	4.08 ± 0.27	3.92 ± 0.15	0.129
PTH (pg/mL)	52.5 (1–203.8)	62 (22.6–154.7)	0.166
Cystatin C (mg/L)	1.26 ± 0.38	1.61 ± 0.41	0.017
NTproBNP (pg/mL)	89.3 (40–1772.3)	88.5 (44.5–1312)	0.953
Microalbuminuria/24 h (mg/24 h)	65 (30.5–283.2)	684.8 (307.4–1804)	0.000
Proteinuria/24 h (mg/24 h)	173.5 (40–432)	624 (435–2419)	0.014
Cr.Cl (mL/dk) (mL/min)	114.12 ± 37.37	93.9 ± 32.1	0.133
eGFR_MDRD_ (mL/min)	91.57 ± 25.67	73.8 ± 21.44	0.832
eGFR_Cock-Croft_ (mL/min)	101.5 (34.71–286.36)	88.45 (48.57–207.01)	0.421
HOMA-IR	3.92 (0.54–20.91)	3.34 (1.78–11.1)	0.753

Mean ± standard deviation; median (min–max).

**Table 3:** Baseline and sixth-month clinical parameters of the microalbuminuria group.

Parameters	Microalbuminuria Group		
	Baseline	Sixth Month	*p*-Value
Gender (F/M)	54 (31/23)	54 (31/23)	1.00
SBP (mmHg)	130 (109–170)	125 (100–190)	0.522
DBP (mmHg)	80 (60–100)	80 (60–100)	0.081
BMI (kg/m^2^)	31.95 (22.60–49.90)	31.3 (21.7–49.3)	0.717
HbA1c (%)	6.7 (5.3–8)	6.8 (5.3–9.5)	0.176
FPG (mg/dL)	117 (72–227)	124.5 (84–277)	0.657
2 h-PG (mg/dL)	173.5 (83–363)	168 (83–385)	0.629
Fasting insulin (µIU/mL)	13 (2.2–61.8)	13.85 (5.1–41.1)	0.061
LDL (mg/dL)	112.5 (53–238)	109 (49–179)	0.917
HDL (mg/dL)	42 (26–68)	43 (26–75)	0.036
TG (mg/dL)	128.5 (35–489)	131 (40–767)	0.725
Cr (mg/dL)	0.85 (0.45–1.87)	0.82 (0.48–1.82)	0.852
ALT (U/L)	19 (8–70)	20 (11–54)	0.815
Vitamin D (µg/L)	16.25 (3.7–66.4)	21.65 (5–51.7)	0.006
Ca (mg/dL)	9.4 (8.5–10.2)	9.3 (8.6–10.2)	0.641
P (mg/dL)	3.71 ± 0.56	3.53 ± 0.58	0.019
Alb (g/dL)	4.08 ± 0.27	4.01 ± 0.46	0.181
PTH (pg/mL)	52.5 (1–203.8)	42.3 (1–202)	0.001
Cystatin C (mg/L)	1.26 ± 0.38	1.27 ± 0.47	0.544
NTproBNP (pg/mL)	89.3 (40–1772.3)	97.95 (43.5–618.7)	0.128
Microalbuminuria/24 h (mg/24 h)	65 (30.5–283.2)	37.4 (5–259)	0.001
Proteinuria/24 h (mg/24 h)	173.5 (40–432)	124.5 (33–595)	0.013
Cr.Cl (mL/min)	114.12 ± 37.37	113.42 ± 46.16	0.491
eGFR_MDRD_ (mL/min)	91.57 ± 25.67	89.68 ± 24.81	0.338
eGFR_Cock Croft_ (mL/min)	101.5 (34.71–286.36)	99.04 (37.69–264.92)	0.664
HOMA-IR	3.92 (0.54–20.91)	3.96 (1.07–14.21)	0.053

Mean ± standard deviation; median (min–max).

**Table 4:** Baseline and sixth-month clinical parameters of macroalbuminuria group.

Parameters	Macroalbuminuria Group		
	Baseline	Sixth Month	*p*-Value
Gender (F/M)	9 (8/1)	9 (8/1)	1.00
SBP (mmHg)	143.13 ± 15.7	142.11 ± 13.9	0.272
DBP (mmHg)	80.63 ± 10.15	77 ± 10.78	0.275
BMI (kg/m^2^)	35.21 ± 5.56	35.43 ± 5.55	0.322
HbA1c (%)	7.24 ± 0.70	7.75 ± 1.07	0.099
FPG (mg/dL)	135.22 ± 32.89	158.78 ± 61.5	0.151
2 h-PG (mg/dL)	229 ± 59.2	228.6 ± 108.9	0.994
Fasting insulin (µIU/mL)	15.48 ± 8.76	21.02 ± 13.6	0.144
LDL (mg/dL)	108 ± 34.42	103.44 ± 37.9	0.678
HDL (mg/dL)	44.66 ± 9.48	45.11 ± 13.82	0.848
TG (mg/dL)	187.77 ± 93.6	197.77 ± 104.7	0.787
Cr (mg/dL)	0.95 ± 0.28	1.04 ± 0.27	0.091
ALT (U/L)	23.66 ± 6.78	23 ± 11.67	0.109
Vitamin D (µg/L)	10.9 ± 3.19	31.37 ± 14.9	0.004
Ca (mg/dL)	9.67 ± 0.389	9.57 ± 0.48	0.340
P (mg/dL)	3.64 ± 0.35	3.32 ± 0.64	0.163
Alb (g/dL)	3.92 ± 0.15	3.81 ± 0.16	0.230
PTH (pg/mL)	62 (22.6–154.7)	50.8 (27.2–165.7)	0.374
Cystatin C (mg/L)	1.61 ± 0.41	1.53 ± 0.27	0.405
NTproBNP (pg/mL)	88.5 (44.5–1312)	145.2 (54.3–1339.6)	0.214
Microalbuminuria /24 h (mg/24 h)	684.8 (307.4–1804)	460 (17.9–952.6)	0.021
Proteinuria/24 h (mg/24 h)	624 (435–2419)	575 (66–1650)	0.028
Cr.Cl (mL/min)	93.9 ± 32.1	90.42 ± 29.86	0.203
eGFR_MDRD_ (mL/min)	73.8 ± 21.44	65.66 ± 17.71	0.028
eGFR_CockCroft_ (mL/min)	103.36 ± 50.28	93.52 ± 42.32	0.046
HOMA-IR	3.34 (1.78–11.1)	7.17 (1.54–19.93)	0.110

Mean ± standard deviation, median (min–max).

## Data Availability

The data presented in this study are available on request from the corresponding author.

## Abbreviations

ACEI	Angiotensin-Converting Enzyme Inhibitors
ADA	American Diabetes Association
Alb	Albumin
ALT	Alanine Aminotransferase
ARB	Angiotensin Receptor Blockers
ASA	Acetylsalicylic acid
BMI	Body Mass Index
Ca	Calcium
CDC	Centers for Disease Control and Prevention
Cr.Cl	Creatinine Clearance
Cr	Creatinine
DBP	Diastolic Blood Pressure
DKD	Diabetic Kidney Disease
DM	Diabetes Mellitus
DN	Diabetic Nephropathy
eGFR	Estimated Glomerular Filtration Rate
FPG	Fasting Plasma Glucose
HDL	High-Density Lipoprotein
IDF	International Diabetes Federation
IGT	Impaired Glucose Tolerance
LDL	Low-density lipoprotein
NTproBNP	N-Terminal Pro B-Type Natriuretic Peptide
OAD	Oral Antidiabetic Drugs
P	Phosphorus
PG	Plasma Glucose
PTH	Parathormone
RAS	Renin–Angiotensin System
SBP	Systolic Blood Pressure
TG	Triglyceride
TGF	Transforming Growth Factor
VDD	Vitamin D Deficiency
VDR	Vitamin D Receptor
